# Pyroptosis-Related Inflammasome Pathway: A New Therapeutic Target for Diabetic Cardiomyopathy

**DOI:** 10.3389/fphar.2022.842313

**Published:** 2022-03-07

**Authors:** Zhengyao Cai, Suxin Yuan, Xingzhao Luan, Jian Feng, Li Deng, Yumei Zuo, Jiafu Li

**Affiliations:** ^1^ Key Laboratory of Medical Electrophysiology, Ministry of Education and Medical Electrophysiological Key Laboratory of Sichuan Province, Department of Cardiology, Institute of Cardiovascular Research, The Affiliated Hospital of Southwest Medical University, Southwest Medical University, Luzhou, China; ^2^ Department of Neurosurgery, The Affiliated Hospital of Southwest Medical University, Luzhou, China; ^3^ Department of Rheumatology, The Affiliated Hospital of Southwest Medical University, Luzhou, China; ^4^ Department of outpatient, The 13th Retired Cadre Recuperation Clinic Of Chengdu, Institute of Cardiovascular Research, Chengdu, China

**Keywords:** pyroptosis, diabetes mellitus, diabetic cardiomyopathy, nod-like receptor protein 3, gasdermine

## Abstract

Pyroptosis is a highly specific type of inflammatory programmed cell death that is mediated by Gasdermine (GSDM). It is characterized by inflammasome activation, caspase activation, and cell membrane pore formation. Diabetic cardiomyopathy (DCM) is one of the leading diabetic complications and is a critical cause of fatalities in chronic diabetic patients, it is defined as a clinical condition of abnormal myocardial structure and performance in diabetic patients without other cardiac risk factors, such as hypertension, significant valvular disease, etc. There are no specific drugs in treating DCM despite decades of basic and clinical investigations. Although the relationship between DCM and pyroptosis is not well established yet, current studies provided the impetus for us to clarify the significance of pyroptosis in DCM. In this review, we summarize the recent literature addressing the role of pyroptosis and the inflammasome in the development of DCM and summary the potential use of approaches targeting this pathway which may be future anti-DCM strategies.

## 1 Introduction

Diabetes mellitus (DM) is a significant public health issue all over the world (Kim, 2018; Rendra et al., 2019). Diabetic complications remain the main cause of morbidity and mortality in diabetic patients, with cardiovascular disease being the leading cause of death. Diabetic cardiomyopathy (DCM) is phenotypically defined as the structural or functional changes of the heart occurring in a diabetic patient independent of other comorbidities such as hypertension, coronary disease, and valvular disease as well as independent of other conventional cardiovascular risk factors, resulting in either the systolic or diastolic dysfunction (Rubler et al., 1972), causing a substantial detriment to the patient’s quality of life (Yap et al., 2019). There are growing lines of evidence indicating myocardial inflammation as a key process in DCM development (Boudina and Abel, 2007; Mann, 2015; Prabhu and Frangogiannis, 2016). Hyperglycemia-induced reactive oxygen species (ROS) generation is considered to be responsible for the progression and development of DCM (Cai, 2006; Cai et al., 2006). The increased ROS could induce many cytokines and inflammatory factors, such as nuclear factor-kB (NF-kB), thioredoxin interacting/inhibiting protein (TXNIP), and inflammasome (Bryant and Fitzgerald, 2009; Devi et al., 2012; Tsai et al., 2012). Although inflammasome was shown to be involved in the pathogenic mechanisms of diabetes and its complications, the potential role and regulatory mechanism of the inflammasome in DCM has remained largely unexplored.

Pyroptosis, an emerging type of programmed cell death (Man et al., 2017; Wang et al., 2018a), is associated with the inflammatory response and is activated by bacteria, pathogens, or their endotoxins, leading to the subsequent activation of the caspase family, accompanied by cell swelling, cell membrane pore formation, cell membrane rupture, inflammasome activation, as well as the release of cell contents and inflammatory mediators, resulting in a robust inflammatory response. In recent years, pyroptosis has gradually become a very important therapeutic target for inflammation. DCM is closely related to chronic inflammation, and accumulating evidence implicated pyroptosis as a critical contributor to myocardial inflammation in DCM (Li et al., 2014; Luo et al., 2014; Luo et al., 2017; Yang et al., 2018a; Yang et al., 2018b). This review focuses on the molecular and pathophysiological mechanisms of the pyroptosis-related inflammasomes pathway in the development of DCM are summarized. With this review, we attempted to provide new insights for researchers regarding the development of potential therapies for DCM.

## 2 Pyroptosis: A Newly DISCOVERED Type of Programmed Cell Death

### 2.1 The Relationship Between Cell Death and Pyroptosis

Cell death is a ubiquitous life phenomenon that is mainly divided into cell necrosis and programmed death, of which programmed death includes apoptosis, autophagy, pyroptosis, and other modes (Soengas et al., 1999; Levine et al., 2011; Strowig et al., 2012; Pasparakis and Vandenabeele, 2015; Wallach et al., 2016). Pyroptosis is a newly discovered type of programmed cell death associated with inflammatory responses. Compared with apoptosis, pyroptosis is characterized by cell swelling, perforation, lysis, and release of cell contents. Pyroptosis occurs in many cell lines including endothelial cells, smooth muscle cells, and cardiac myocytes, and it is widely involved in the pathophysiological processes of various diseases (Pan et al., 2018; Yu et al., 2018). Pyroptosis is mediated by numerous inflammasomes that can detect exogenous or endogenous danger signals and is characterized by activation of nod-like receptor protein-3 (NLRP3) inflammasome and caspase and the release of interleukin (IL)-1β and IL-18 (Xia et al., 2019; Yu et al., 2020), thereby playing a critical role in multiple inflammatory and immune-mediated diseases (Yang et al., 2014; Wang et al., 2018b; Johnson et al., 2018; Wu et al., 2019). Interestingly, pyroptosis is like a “double-edged sword” in cell function. On one hand, it rapidly eliminates intracellular pathogens by coordinating antimicrobial host defenses (Kleinbongard et al., 2018) and helps protect multicellular organisms from bacterial infection. On the other hand, uncontrolled pyroptosis will result in a severe impact on cellular environmental homeostasis through pathological and inflammatory cascades (Shi et al., 2017), finally leading to chronic low-grade inflammation. This contradiction may be attributed to differences in the virulence strategies used and the cell types targeted by different pathogens (Miao et al., 2010).

In 2015, two studies published in Nature identified gasdermin D (GSDMD) as a substrate for inflammatory caspases and showed that it was essential for inflammatory caspases-dependent pyroptosis and IL-1β secretion (Kayagaki et al., 2015; Shi et al., 2015). In 2018, the Nomenclature Committee on Cell Death redefined pyroptosis as a programmed death of plasma membrane pores formed by GSDM protein family members, which is an inflammatory reaction, often (but not always) as a consequence of inflammatory caspase activation (Galluzzi et al., 2018). recent studies have shown that proteolytic activation of GSDME, GSDMB, and GSDMC by certain caspases and granzymes can lead to pyroptosis (Rogers et al., 2017; Wang et al., 2017; Broz et al., 2020; Hou et al., 2020; Zhang et al., 2020; Zhou et al., 2020). GSDM members can be cleaved by a variety of proteases that are activated or inactivated, and most of the proteases that induce pyroptosis can also induce apoptosis in the absence of the corresponding GSDM protein, which means that GDSM can convert apoptosis into pyroptosis (Table 1).

**TABLE 1:** Discrimination between apoptosis and pyroptosis.

	Apoptosis	Pyroptosis
Common point	Programmed cell death	
Characteristic	Cell shrinkage	Cell enlarging
	Membrane blebbing	Membrane broken
	Nuclear DNA fragmentation	Organelles deforming
	Nuclear condensation	DNA randomly degraded
	—	Chromatin condensation
Dependence on caspases Transformation to apoptosis and pyroptosis	Caspase 3, 6, 7, 8,9, 10	Caspase 1, 3, 4, 5, 11
	caspases that activate GSDM members: ①GSDMC by caspase-8 Watabe et al. (2001); ②GSDMD by caspase-1, and to a lesser extent, by caspase-8 Orning et al. (2018), Sarhan et al. (2018); ③GSDME by caspase-3 Rogers et al. (2017), Wang et al. (2017)	

### 2.2 Types and Processes of Pyroptosis

**FIGURE 1:**
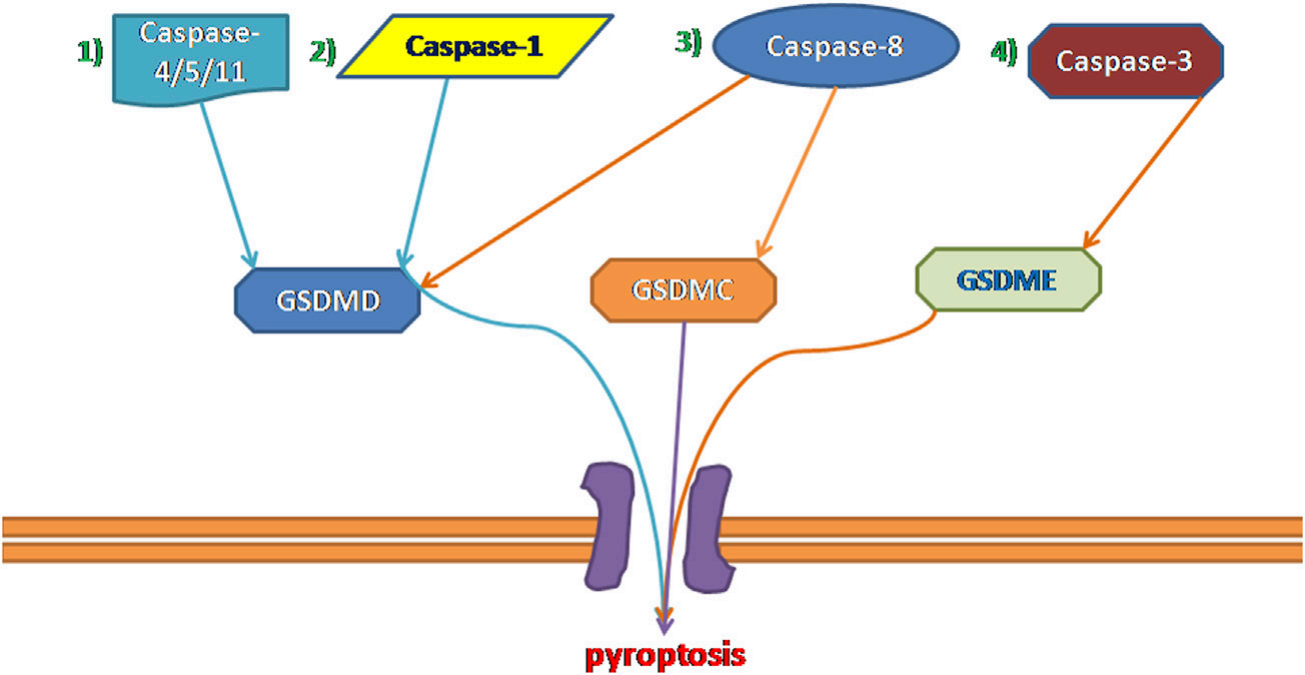
Four types of pyroptosis: 1) Non-canonical Inflammasome Pathways Associated With Pyroptosis. 2) canonical Inflammasome Pathways Associated With Pyroptosis. 3) Caspase-8-dependent pyroptosis pathway. 4) Caspase-3-dependent pyroptosis pathway.

Inflammatory caspases cleave the GSDM protein to trigger pyroptosis and result in pore formation in the membrane, the release of proinflammatory cytokines, and, finally, programmed cell death (Yuan et al., 2016). Currently, pyroptosis can be divided into four types (Figure 1) according to different initiate activation modes, namely classical pyroptosis pathway, nonclassical pyroptosis pathway, caspase-3-dependent pyroptosis pathway, and caspase-8-dependent pyroptosis pathway. Notably, in humans, GSDM family members are composed of six members: GSDMA, B, C, D, E, and Pejvakin, and all have a highly conserved N-terminal domain that induces pyroptosis when expressed ectopically, except for PJVKNT (Broz et al., 2020). For example,caspase-3 and caspase-8 can induce pyroptosis via the cleavage of GSDME and GSDMD, respectively (Kayagaki et al., 2015; Jorgensen et al., 2017; Wang et al., 2017). Although these four types have their characteristics, they are related to each other. In addition, they share a common endpoint event which is to process IL-18 and IL-1β, activate the perforating protein GSDMD, and eventually cause the cell membrane to break and release IL-18 and IL-1β (Ding et al., 2016; Kovacs and Miao, 2017).

#### 2.2.1 Canonical Inflammasome Pathway Associated With Pyroptosis

Under the stimulation of dangerous signals, cysteinyl aspartate specific protease-1 (caspase-1) is activated by the assembled and activated inflammasome complex; this induces further cell membrane degradation, leading to cell death and release of mature IL-1β and mature IL-18 (Russo et al., 2016; Błażejewski et al., 2017). This caspase-1-dependent pyroptosis is known as the canonical inflammasome pathway. The specific steps are as follows:

The cells activate their respective inflammasomes, including NLRP3, absent in melanoma 2, or pyrin through the action of pathogen-associated molecular patterns and danger‐associated molecular patterns under the stimulation of various external factors, such as hyperglycemia, inflammation, and hyperlipidemia. The activation of NLRP3 initiates pro-caspase-1 self-cleavage to form a caspase-1 mature body. On the one hand, activated caspase-1 recognizes inactive IL-β and IL-18 precursors and converts them into mature inflammatory cytokines. On the other hand, caspase-1 cleaves GSDMD that mediate the formation of membrane pores. The formation of membrane pores promotes the release of inflammatory factors, cell swelling, and finally, pyroptosis (Afonina et al., 2017; Man et al., 2017).

#### 2.2.2 Non-Canonical Inflammasome Pathways Associated With Pyroptosis

In 2011, Kayagaki et al. (2011) discovered non-canonical pyroptotic pathways. In contrast to canonical pyroptotic pathways, the cell wall LPS of Gram-negative bacteria bypasses TLR4 and directly combines with the pro-caspase (-4 and -5 in humans and -11 in murine) to form activated caspase-4/5/11, and then cleaves the 53-kDa precursor form of GSDMD (pro-GSDMD) to generate N-terminal of mature GSDMD p30 fragment (Kayagaki et al., 2015), which further causes membrane pore formation, the release of IL-1b and IL-18 in the cell and induces pyroptosis (Hagar et al., 2013; Shi et al., 2014; Shi et al., 2017). This pathway does not involve caspase-1; in the absence of caspase-1, human caspase-4/5 and murine caspase-11 can also induce pyroptosis with all associated morphological characteristics (Broz et al., 2020). Although the activation pathways are different, the downstream signaling pathways are all activated caspases that cleave GSDMD and release the N-terminal domain to form membrane pores, eventually leading to pyroptosis. In other words, GSDMD is a necessary downstream component of both the canonical and non-canonical inflammasome pathways associated with pyroptosis (Kayagaki et al., 2015; Shi et al., 2015; Aglietti et al., 2016; Liu et al., 2016; Sborgi et al., 2016). However, the current understanding of the noncanonical caspase-11/4/5 pathway mainly focuses on its role in infectious diseases (Shi et al., 2014; Yi, 2017). The role of caspase-11/4/5 in cardiovascular disease is rarely reported; this may be a research direction in the future cardiovascular field.

#### 2.2.3 Caspase-3-Dependent Pyroptosis Pathway

Caspase-3 is traditionally only induced apoptosis, however, recent studies have shown that caspase-3 also plays an important role in pyroptosis. Due to the presence of a natural caspase-3 cleavage site in the N- terminal and C- terminal structural domains of GSDME, activated caspase-3 is capable of cleaving a specific site of GSDME to release an active N- terminal domain and perforating the plasma membrane to induce pyroptosis (Rogers et al., 2017). Another study has recently also reported that GSDME has the same function as GSDMD, and can also activate the intrinsic pathway downstream of inflammasome activation (Rogers et al., 2019). Briefly, GSDME is activated by caspase-3 to further generate the GSDME-N fragments and then cause the pore-forming effect of the cell membrane to mediate pyroptosis. However, the study of caspase-3 induced pyroptosis is still very limited, and future studies should focus on the mechanism of caspase-3 induced pyroptosis.

#### 2.2.4 Caspase-8-Dependent Pyroptosis Pathway

As mentioned above, the cleavage of GSDMD and membrane pore formation is key to pyroptosis, a recent study by Orning et al. showed that recombinant mouse GSDMD is cleaved by purified active caspase-8 (Orning et al., 2018), although it was less efficient at GSDMD processing in comparison with caspase-1, its activity was sufficient to trigger pyroptotic cell death in murine macrophages. In recent years, many independent studies have revealed synchronicity between caspase-8 activity and GSDMD-mediated pyroptosis in multiple scenarios. In Chen/Demarco (Chen et al., 2019) et al.'s study, they found that the Caspase-8 activity led to cleavage at position D276, the cleavage site used by caspase-1 generating the p30 fragment, which means that caspase-8 generated the same pyroptosis-mediating p30 fragment as caspase-1. Various signs suggest that there may be a close relationship between caspase-8 and pyroptosis, but given that not enough attention has been paid to caspase-8 by scholars, the mechanism by which caspase-8 induced pyroptosis needs to be further explored.

### 2.3 Diabetes and Pyroptosis

Although the pathogenesis of type 1 diabetes (T1DM) and type 2 diabetes (T2DM) is differentiated, studies have found that they are each closely related to pyroptosis. Generating a T1DM model is to induce pancreatic damage using streptozotocin (STZ) while the most usual approach to developing a T2DM model is to feed animals with a high-fat diet (HFD). Previously, it was believed that the pathogenesis of T1DM involved adaptive immunity mediated by T cells. However, an increasing number of studies have shown that the Toll-like receptor (TLR)-mediated innate immune system also plays an important role in the pathogenesis of T1DM (Needell and Zipris, 2017). Carlos et al. confirmed that NLRP3-dependent IL-1 β production mediated by mDNA leads to T1DM. The important pathological features of T2DM are insulin resistance and impaired insulin secretion from pancreatic β-cells. Insulin resistance is closely associated with inflammation. Numerous evidence suggests elevated expression of inflammasome components (NLRP3 caspase-1 and ASC) in untreated T2DM patients (Joya-Galeana et al., 2011; Lee et al., 2013). And the secretion of IL-1β and IL-18, caused by activation of the NLRP3 inflammasome, is emerging as a powerful determinant of metabolic inflammation and insulin resistance in T2DM patients (Stienstra et al., 2010; Wen et al., 2011). Consistent with these findings, higher serum levels of IL-1β and IL-18 have been reported in drug-naïve T2DM patients compared with healthy subjects (Lee et al., 2013). Under a hyperglycemic environment, ROS activates the NLRP3 inflammasome in β cells, elevating caspase-1-dependent IL-1β secretion; this mediates dysfunction of β-cell insulin secretion and promotes obesity and insulin resistance, finally leading to pyroptosis and the development of T2DM.

### 2.4 Pyroptosis and Diabetic Cardiomyopathy

Pyroptosis is triggered by various pathological stimuli, such as oxidative stress, hyperglycemia, inflammation, and it is crucial for controlling microbial infections. It was first identified in the macrophage in 1992, which presented rapid lysis after infection with Shigella flexneri, (Cookson and Brennan, 2001)and the name was coined in 2001 (Cookson and Brennan, 2001). Pyroptosis is a highly regulated cell death process, and it plays a pivotal role in the pathogenesis of various cardiovascular diseases (CVDs) such as myocardial infarction (Gonzalez-Pacheco et al., 2017; Yang et al., 2017),hypertension (Bruder-Nascimento et al., 2016; Zhu et al., 2017), and cardiomyopathy (Pereira et al., 2018), and involves endothelial cells (Zhang et al., 2018a), VSMCs (Pan et al., 2018) and so on. Therefore, this process is a potential target for therapeutic intervention to prevent CVDs.

Today, increasing evidence suggests that pyroptosis is involved in the pathogenesis of cardiomyocyte injury, especially in DCM (Qiu et al., 2017). DCM, a complication of diabetes, is characterized by myocardial fibrosis, left ventricular hypertrophy, and damaged left ventricular systolic and diastolic function (Jia et al., 2018a). Inflammation is implicated in the pathogenesis of diabetic cardiomyopathy (Zhang et al., 2017). Oxidative stress, coupled with the activation of downstream pro-inflammatory and cell-death pathways, induces DCM-associated pathological changes (Althunibat et al., 2019). Therefore, anti-inflammatories may be useful for the prevention and treatment of diabetic complications. In recent years, the role of the NLRP3 inflammasome in diabetic cardiomyopathy has drawn much attention. And NLRP3 also plays an important role in the development of pyroptosis. By activating NLRP3, stimulates the production of IL-1β and IL-18 (Zeng et al., 2017; Ge et al., 2018), triggers pyroptosis, and ultimately leads to the development of diabetic cardiomyopathy.

Due to the different modeling methods of T1DM and T2DM, the pathophysiological mechanism of DCM caused by them will also change accordingly (Hölscher et al., 2016). Insulin may be one of the reasons for this difference. Insulin signaling in two types of diabetes is very different. T1DM is characterized by insulin deficiency, and T2DM is characterized by insulin resistance. However, recent studies have shown that no matter what type of diabetic cardiomyopathy leads to diabetes, the final manifestation is cardiac diastolic dysfunction (Hölscher et al., 2016). Although the etiology of these two types of diabetes is different, there are still some common molecular changes in the myocardium (Hölscher et al., 2016). In both two types of diabetes, proper glycemic control (Salvatore et al., 2021) is a key factor to prevent DCM progression from heart failure.

What’s more, In the diabetic heart, the NLRP3 inflammasome responds to hyperglycemia-induced toxicity and initiates the progression of pyroptosis (Luo et al., 2014; Luo et al., 2017; Zhou et al., 2018). In recent years, a growing body of evidence suggests that inhibition of the NLRP3 inflammasome may slow pyroptosis in diabetes and associated complications (Yang et al., 2018b; Wu et al., 2018; Song et al., 2019). But there remain some problems, though DCM is a common clinical complication in patients with diabetes, there are few studies about the mechanism between DCM and pyroptosis, and the mechanism of how to activate NLRP3 is still not clear too.

## 3 Signaling Pathways Related to the Pyroptosis of Diabetic Cardiomyopathy

Mitochondrial ROS have a central role in NLRP3 inflammasome activation (Zhou et al., 2011; Zhong et al., 2013). One study demonstrated that the production of intracellular ROS induces NLRP3 translocation to the cytoplasm from the nucleus in LPS treated neonatal rat cardiomyocytes. And NLRP3 cytoplasmic translocation can be prevented by the elimination of ROS (Li et al., 2019a). And the accelerated ROS production induced by high glucose plays a key role in the progression of diabetic cardiovascular disease and cardiomyocyte pyroptosis (Chen et al., 2017). A recent study showed that Gypenosides, a traditional Chinese medicine, can reduce activation of the NLRP3 inflammasome by inhibiting ROS production, and this can improve damage to the myocardium induced by high glucose (Zhang et al., 2018b). And GSDMD cleavage occurs downstream of ROS release. In conclusion, excessive generation of ROS and NLRP3 inflammasome activation trigger inflammation and pyroptosis in diabetes. But the specific mechanism by which NLRP3 triggers anxiety in DCM is still unclear.

### 3.1 TLR4/NF-kB/NLRP3 Inflammasome Signaling Pathway

Toll-like receptor 4 (TLR4), Myeloid differentiation factor 88 (MyD88), and nuclear factor kappa-light-chain- enhancer of activated B cells (NF-κB) pathway contribute to NLRP3 inflammasome activation (Bauernfeind et al., 2009). NF-κB is closely associated with NLRP3 and plays a crucial part in the regulation of genes involved in immunity and inflammation (Sun, 2017). On the one hand, NF-κB binds to the NLRP3 promoter region and affects transcriptional regulation of NLRP3 (Qiao et al., 2012). On the other hand, blockage of NF-κB exacerbates the activation of the NLRP3-dependent inflammasome (Afonina et al., 2017). Then, NLRP3 inflammasome forms a complex with its adaptor apoptosis-associated speck-like protein containing a CARD (ASC) which leads to the enhancement of pro-caspase-1 and the formation of an active caspase-1 (Latz et al., 2013). The activated caspase-1 converts pro-IL-1β and IL-18 into its mature forms and then induces pyroptosis (Shi et al., 2015; Youm et al., 2015; Sanchez-Lopez et al., 2019). This process has a protective effect during the initial inflammation. Nevertheless, when IL-1β and IL-18 are continually released and accumulated in the cell, they induce pyroptosis, tissue damage, and organ dysfunction (Green et al., 2018). The above idea was demonstrated in the rat model of Hepatic ischemia/reperfusion injury by Alaa El-Din El-Sayed (El-Sisi et al.,2021; Zhang et al., 2021) and in the premature ovarian failure model made by Cairong Zhang et al. (2021). Moreover, Wang Y. et al. found that chemical GSDMD-related pyroptosis of tubular cells in diabetic kidney disease is dependent on the TLR4/NF-kB signaling pathway (Wang et al., 2019). In summary, we speculate that pyroptosis can be associated with DCM through the TLR4/NF-kB/NLRP3 Inflammasome Signaling Pathway, but there are very few scholars studying this pathway in DCM, and future directions can focus on the understanding of this pathway.

### 3.2 AMPK/ROS/Thioredoxin-Interacting Protein (TXNIP)/NLRP3 Inflammasome Signaling Pathway

In recent years, TXNIP has been recognized as a central contributor to diabetic vascular complications (Li et al., 2017; Lu et al., 2018; Gu et al., 2019). TXNIP levels will be increased in hyperglycemic cultured cells as well as in peripheral blood and tissues of diabetic animals (Li et al., 2017; Gu et al., 2019). The TXNIP/NLRP3 pathway was activated due to increased ROS production induced by high glucose. All NLRP3 agonists trigger the production of ROS, which leads to activation of the NLRP3 inflammasome via the ROS-sensitive TXNIP protein (Schroder et al., 2010). Mitochondrial dysfunction can produce a large number of reactive oxygen species (ROS), and this, in turn, induces dissociation of TXNIP and thioredoxin. TXNIP then binds to NLRP3 through the leucine-rich repeat domain, prompting activation of the NLRP3 inflammasome (Strowig et al., 2012; Qiu and Tang, 2016; Han et al., 2018).

TNXIP is a potential regulator involved in high glucose-induced cardiomyocytes. A recent study showed that glucose-treated H9c2 cardiomyocytes produced excessive ROS in a concentration-dependent manner, and the level of TXNIP showed a similar expression pattern in response to glucose (Bauer et al., 2014). Recently, the role of AMPK on TNXIP has also gradually attracted attention. The activation of AMPK is driven by oxidative stress via ROS-dependent phosphorylation (Mungai et al., 2011). Previous studies revealed that AMPK is a key regulator of energy metabolism and inflammation in DCM (Jia et al., 2018a; Jia et al., 2018b). In high glucose-treated cardiomyocytes, a slight increase in phosphorylated AMPK was observed. Activated AMPK degenerates TXNIP to manipulate the activity of the NLRP3 inflammasome (Wu et al., 2013). What’s more, in experiments by Wei H et al., they also confirmed an anti-pyroptotic pathway mediated by the ROS-AMPK-TXNIP pathway, which regulates the activity of inflammasome and caspase-1 in diabetic cardiomyocytes. In summary, the ROS-AMPK-TXNIP pathway can serve as a link between oxidative stress and cardiac inflammation in various CVDs (Wei et al., 2019).

### 3.3 AMPK/SIRT1/Nrf2/HO-1/NF-kB Inflammasome Signaling Pathway

SIRT1 is a member of the sirtuin family, which is involved in many diseases and bioprocesses such as cancer development, oxidative stress, and pyroptosis (Simic et al., 2013; Ma et al., 2016; Qu et al., 2017; Chen et al., 2018). The AMPK/SIRT1 pathway could modulate the function of vascular endothelial cells in diversiform ways, for instance, activating PGC-1α, Nrf2, and FoxO3 and inhibiting the activity of various inflammation-related proteins such as p38MAPK and NF-κB pathway. Endothelial dysfunction is closely related to DCM, so we supposed that the AMPK/SIRT1 pathway plays an important role in the pathological progress of DCM.

As one of the most essential transcription factors, nuclear factor erythroid 2-related factor 2 (Nrf2) exerts antioxidant, anti-apoptotic, and anti-inflammatory effects by interacting with multiple signaling pathways (Loboda et al., 2016; Hao et al., 2019), with an important role in cytoprotection, is activated under stress conditions when excessive ROS is detected (Tsushima et al., 2019; Ungvari et al., 2019). Many studies are showing that SIRT1/PGC-1α/Nrf2 signaling can regulate both pyroptosis and oxidative stress in different situations such as cancer or liver oxidative stress (Do et al., 2014; Zhao et al., 2019). The Nrf2/HO-1 pathway has garnered increased interest (Wardyn et al., 2015). Importantly, in Hao Li et al.'s research, they found that in diabetic cardiomyopathy, piceatannol alleviates inflammation and oxidative stress by activating the Nrf2/HO-1 pathway while inhibiting NF-κB activation in Rat H9C2 cardiac myoblasts. The same phenomenon was found in human umbilical vein endothelial cells by Tang, Qian et al. Knockdown of Nrf2 suppressed enhancement of HO-1 expression and abolished the anti-inflammatory effects (Wardyn et al., 2015). Nrf2 is one of the upstream targets of inflammation induced by NF-κB. Besides, studies also showed that activating the Nrf2 signaling pathway could inhibit NLRP3 inflammasome-dependent pyroptosis in vascular endothelial cells (Li et al., 2019b). Nrf2 is one of the upstream targets of inflammation induced by NF-κB. Therefore, it is not difficult for us to deduce that AMPK/SIRT1/Nrf2/HO-1/NF-kB Inflammasome Signaling Pathway plays an important role in the anxiety process of diabetic cardiomyopathy.

### 3.4 Other Signaling Pathways

In recent years, some other pathways related to the pyroptosis of DCM. have been discovered. 1) FoxO3a/ARC/caspase-1 Signaling Pathway: FoxO3a has been reported to inhibit cell death by targeting its downstream protein ARC in glucose-treated cardiomyocytes (Li et al., 2014). 2) AMPK/mTOR/autophagy pathway: Yang F et al. demonstrated that metformin can suppress the NLRP3 inflammasome through the AMPK/mTOR/autophagy pathway (Yang et al., 2019a). 3) Kcnq1ot1/miR-214-3p/caspase-1 pathway: The long non-coding RNA Kcnq1ot1 was overexpressed in the serum of diabetic patients, as well as in HG-treated cardiac fibroblasts and cardiac tissue of diabetic mice. Kcnq1ot1 targeted caspase-1 and regulated the expression of NLRP3 and its downstream inflammatory cytokines by sponging miR-214-3p. Silencing Kcnq1ot1 inhibited the miR-214-3p/caspase-1 pathway to relieve pyroptosis in DCM models, and ameliorate cardiac function and fibrosis *in vivo* (Yang et al., 2018b). Interestingly, a novel circular RNA, named caspase-1-associated circRNA (CACR), also promotes caspase-1 expression by targeting miR-214-3p, thus inducing pyroptosis in HG-treated cardiomyocytes (Yang et al., 2019b). However, there are few studies on the above pathways in diabetic cardiomyopathy, and the specific mechanism needs to be further explored.

## 4 Discussion

Pyroptosis is a new mode of programmed death, which is closely related to the inflammatory response and has been a research hotspot in recent years. Previous studies have focused on demonstrating the relationship between pyroptosis and various diseases, but there is a lack of research on specific mechanisms, and even if there are specific mechanisms of research, few researchers integrate these mechanisms, which is the original intention of our writing of this paper. As mentioned above, pyroptosis is closely related to the inflammatory response, and DCM can be classified as a type of chronic inflammatory disease, and the current study also shows that there is a close link between DCM and pyroptosis. So, it will be of interest if the relationship between DCM and pyroptosis can be studied in-depth and the development of DCM can be inhibited by regulating some of these molecular mechanisms.

**FIGURE 2:**
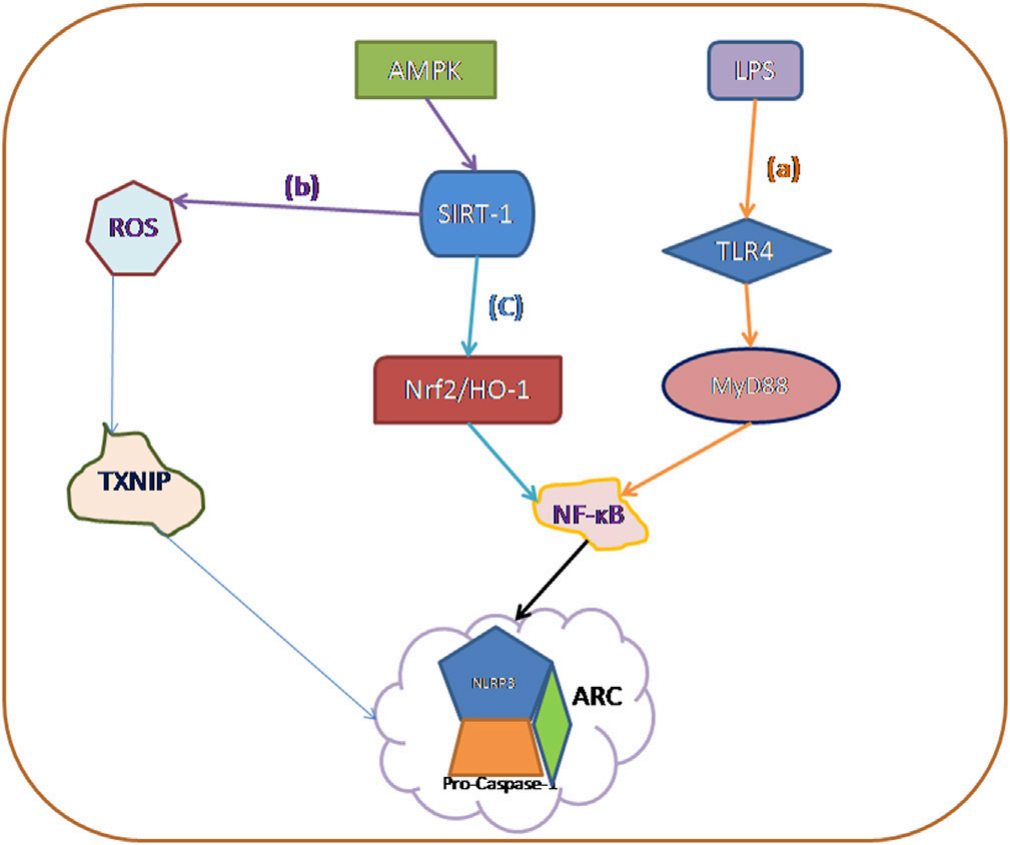
The main activation mechanisms of various caspase families currently involved in pyroptosis: **(A)** TLR4/NF-kB Inflammasome/NLRP3 Inflammasome Signaling Pathway. **(B)** AMPK/ROS/Thioredoxin-Interacting Protein (TXNIP)/NLRP3 Inflammasome Signaling Pathway. **(C)** AMPK/SIRT1/Nrf2/HO-1/NF-kB Inflammasome Signaling Pathway.

In this review, we describe in detail the four ways to trigger pyroptosis, which were previously three, and now with the deepening of research, new mechanisms have emerged, and perhaps in the future, we will find more mechanisms to trigger pyroptosis. Then we summarized three main signaling pathways that currently trigger diabetic cardiomyopathy (Figure 2): 1) TLR4/NF-kB Inflammasome/NLRP3 Inflammasome Signaling Pathway; 2) AMPK/ROS/TXNIP/NLRP3 Inflammasome Signaling Pathway; and 3) AMPK/SIRT1/Nrf2/HO-1/NF-kB Inflammasome Signaling Pathway, which can be used to design DCM-related drugs through the above signaling pathways in the future.

However, through our study, it is found that there are still the following problems to be solved in this direction of DCM-induced pyroptosis: Firstly, the detailed mechanism underlying the function of the gasdermin family in DCM in the downstream pathway of pyroptosis remains unclear. Secondly, small-molecule inhibitors targeting TLR4, NLRP3, and other inflammatory components are potential therapeutic options for DCM. However, there are still many unknown pathways and targets, and corresponding inhibitors, related to the occurrence and development of DCM related to pyroptosis awaiting further exploration. These insights may provide research ideas for developing new mechanisms, drugs, and technologies for DCM. Based on the current summary, we propose the following research targets.

First, the mechanism of pyroptosis triggered by diabetic cardiomyopathy proposed in this review needs more experiments to verify its feasibility. And the independent dependence of each pathway has not been explored, which provides a direction for future studies.

Second, pyroptosis is a double-edged sword, and most of the current research focuses on its bad side. Can we use the advantages of pyroptosis for DCM?

Finally, more attention should be paid to the pathophysiology of DCM, and to understand the possible potential pathways of the pyroptosis-related inflammasome, which can offer new methods and technologies for the clinical treatment of DCM.

## Abbreviations

CVDs, Cardiovascular diseases; caspase-1, Cysteinyl aspartate specific protease-1; DM, Diabetes mellitus; DCM, Diabetic cardiomyopathy; GSDM, Gasdermine; NLRP3, NOD-like receptor protein 3; Nrf2, Nuclear factor erythroid 2-related factor 2; NF-kB, Nuclear Factor-kB; ROS, Reactive Oxygen Species; TXNIP, Thioredoxin interacting/inhibiting protein; TLR4, Toll-like receptor 4; T1DM, Type 1 diabetes; T2DM, Type 2 diabetes.
