# Supplementary figures and images for: Pyroptosis-Related Inflammasome Pathway: A New Therapeutic Target for Diabetic Cardiomyopathy
Source: Front Pharmacol. 2022 Mar 7;13:842313. doi: 10.3389/fphar.2022.842313 (PMC8959892; doi:10.3389/fphar.2022.842313)

## Slide 1
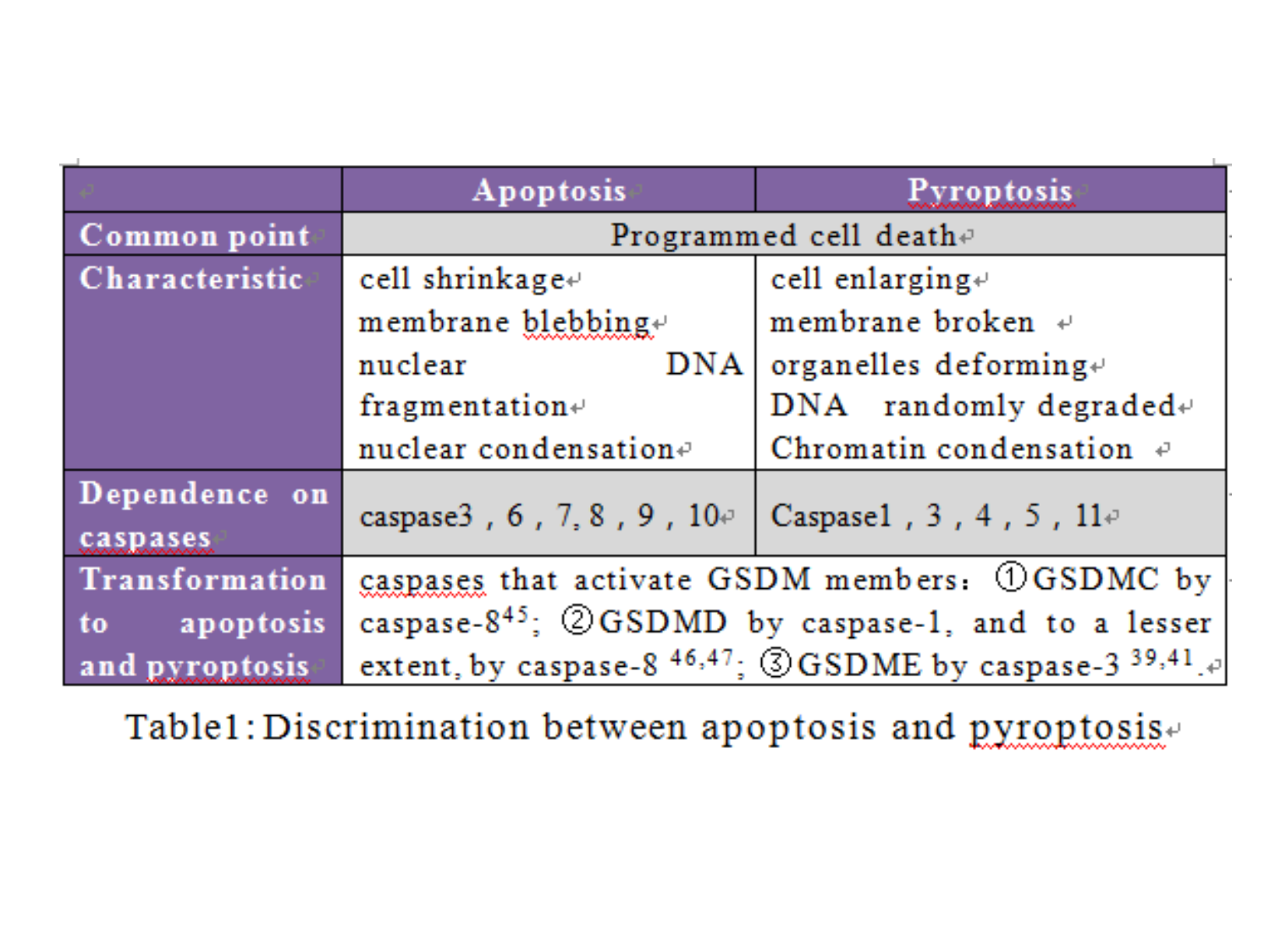

Supplement: Supplementary file 1 [file Presentation1.PPTX]
